# Supplementary material for: Efficiency Enhancement Strategies for Stable Bismuth-Based Perovskite and Its Bioimaging Applications
Source: Int J Mol Sci. 2023 Mar 1;24(5):4711. doi: 10.3390/ijms24054711 (PMC10002936; doi:10.3390/ijms24054711)
Supplement: Supplementary file 1 [file ijms-24-04711-s001.zip › ijms-2222819-supplementary.pdf]

Supplementary material

Efficiency Enhancement Strategies for Stable Bismuth-based Perovskite and Its Bioimaging Applications

Liangyan Xiao[1], Linwei Huang[1], Weihaojia Su[1], Tianjun Wang[2], Haiying Liu[1], Zhongchao Wei[1], Haihua Fan*[1]

1：Guangdong Provincial Key Laboratory of Nanophotonic Functional Materials and Devices, School of Information and Optoelectronic Science and Engineering，South China Normal University，Guangzhou 510006, China

2：School of Biological Science and Medical Engineering，Southeast University，Nanjing 210096, China

S1. Synthesis of Quantum Dots

S1.1 Reagents

All reagents below were not purified, including $BiCl_3$ (99.9%, Macklin), CsCl (99.5%, Macklin), $CeCl_3$ (99.9%, Macklin), oleic acid (OA, AR, Macklin), alcohol anhydrous, dimethyl formaldehyde sulfoxide (DMSO, 99.8%, Macklin), gamma-butyrolactone (AR, Macklin), n-octylamine (AR, Macklin)

S1.2 Preparation of $Cs_3Bi_2Cl_9$ quantum dots

0.2 mmol CsCl (0.04256 g) and 0.134 mmol $BiCl_3$ (0.060121 g) were dissolved in a mixed solution consisting of 1.0 mL DMSO and 2.0 mL GBL, and 35 μL octylamine was added, followed by sonication for 2 h and allowed to settle naturally to obtain the precursor solution. 0.5 mL precursor solution was slowly dropped into the anti-solvent

of anhydrous ethanol (5.0 mL) containing 0.625 mL OA under high-speed stirring, the reaction was continued at 75 °C with slow stirring for 10 min, Finally, centrifuged at 8000 rpm for 10 min to obtain a colorless and clear $Cs_3Bi_2Cl_9$ colloidal solution. The $Cs_3Bi_2Cl_9$ powder was obtained by gradient centrifugation at 5000 rpm and 9000 rpm (10 min per speed) and washed with absolute alcohol at least 3 times, after drying in a drying oven at 75 °C for 6 h.

S1.3 Preparation of $Cs_3Bi_2Cl_9$:Ce quantum dots

The preparation method of the precursor is the same as that of $Cs_3Bi_2Cl_9$ quantum dots (QDs). After obtaining the precursor solution, add $CeCl_3$ with different doping concentration to anhydrous ethanol (5.0mL) containing 0.7mL oleic acid (OA). Note that the concentration refers to experimental use. The ratio of the molar amount of Ce^{3+} used to the total molar amount of (Ce^{3+}+ Bi^{3+}), taking the doping concentration of 30% as an example, that is, n($CeCl_3$) / n($CeCl_3$+$BiCl_3$) = 0.3. Preheat at 80 °C and stir for a certain period of time until the rare earth is completely dissolved. Take 0.5 mL of the precursor solution and drop it very slowly into the anti-solvent stirred at a high speed, seal it with a lid, and continue the reaction at 80 °C with slow stirring for 10 min. A colorless and clear colloidal solution was obtained by centrifugation at 8000 rpm for 10 min. The powder was obtained by gradient centrifugation at 5000 rpm and 9000 rpm (10 min per speed) and washed with absolute alcohol at least 3 times, after drying at 75 °C for 6 h in a drying cabinet.

S1.4 Relative PLQY of $Cs_3Bi_2Cl_9$:Ce QDs made in different conditions. For each trial, the PLQY was normalized to the batch that yields the highest PLQY.

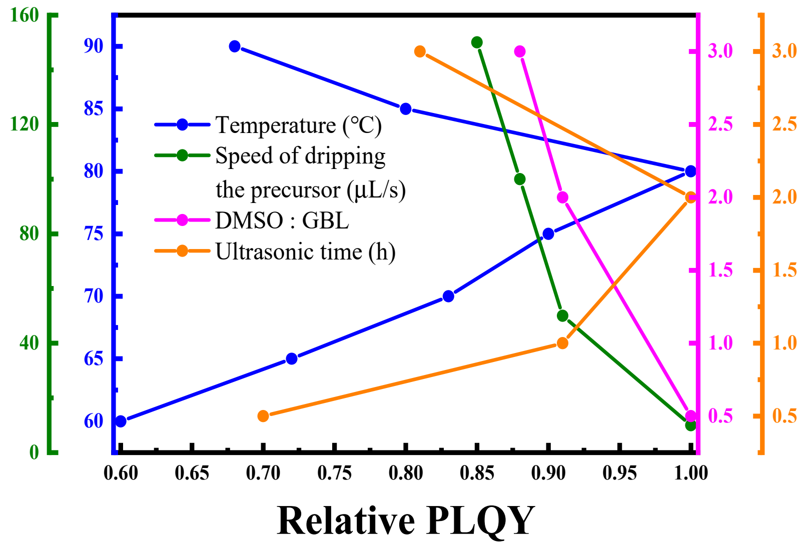

Figure S1. relative PLQY of $Cs_3Bi_2Cl_9$: Ce QDs made in different conditions.

S1.5 Optical property of $Cs_3Bi_2Cl_9$ quantum dots and $Cs_3Bi_2Br_9$ quantum dots

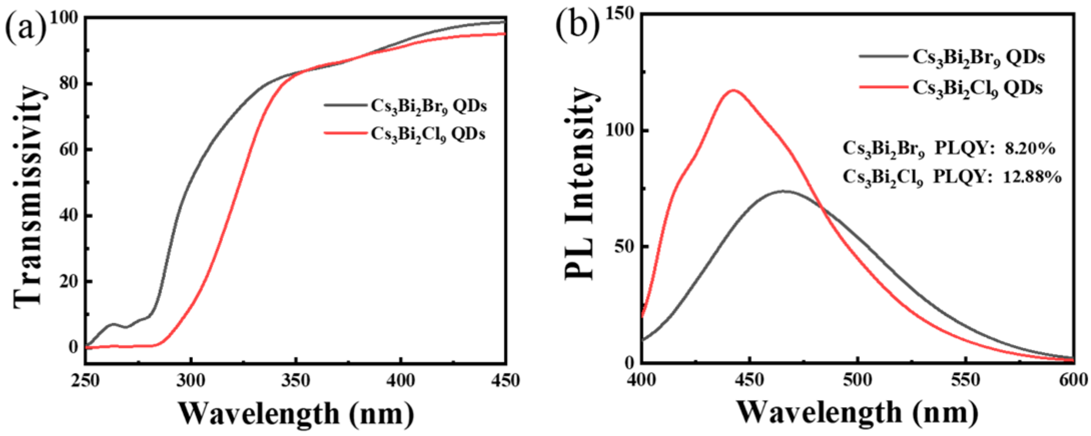

Figure S2. (a) the transition spectra of $Cs_3Bi_2Cl_9$ QDs and $Cs_3Bi_2Br_9$ QDs; (b) the fluorescence spectra of the $Cs_3Bi_2Cl_9$ QDs and $Cs_3Bi_2Br_9$ QDs, the excitation wavelength is 365nm.

S2. Structure Characterization

Transmission electron microscopy (TEM) measurements were performed on a transmission electron microscope (JEM-2100, JEOL Ltd). The phase characterization of the powder samples was carried out using an X-ray diffractometer (BRUKER D8

ADVANCE), the X-ray source was Cu Kα rays (λ = 1.5406Å), the tube current was 40 mA, and the tube voltage was 40 kV. The X-ray Photoelectron Spectroscopy of the powder samples was performed using an X-ray photoelectron spectrometer (AXIS SUPRA, Shimadzu Corporation), X-ray source: Al Kα, and the resulting energy spectrum was calibrated using C 1s (284.6 eV). Plasma emission spectrometer (ICAP-qc, Thermo Fisher, Germany) was used to calibrate the element content. The elements to be measured were Bi, Ce, Cl, and the Cs element content could not be accurately determined, so it was not considered. Raman spectroscopy was performed on a Renishaw in Via confocal Raman microscope with an excitation wavelength of 785 nm.

S2.1 XRD powder diffraction patterns of samples with different doping concentrations

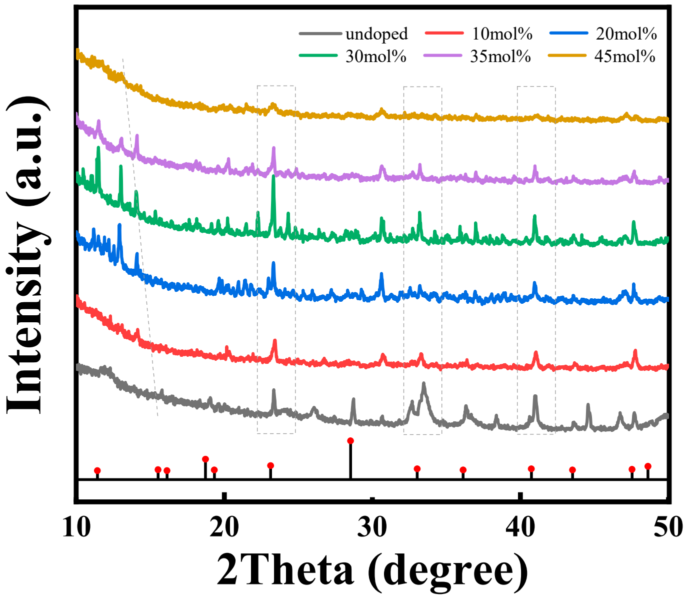

Figure S3. XRD pattern of powder sample.

S2.2 TEM images of $Cs_3Bi_2Cl_9$ and $Cs_3Bi_2Cl_9$: Ce 30% QDs

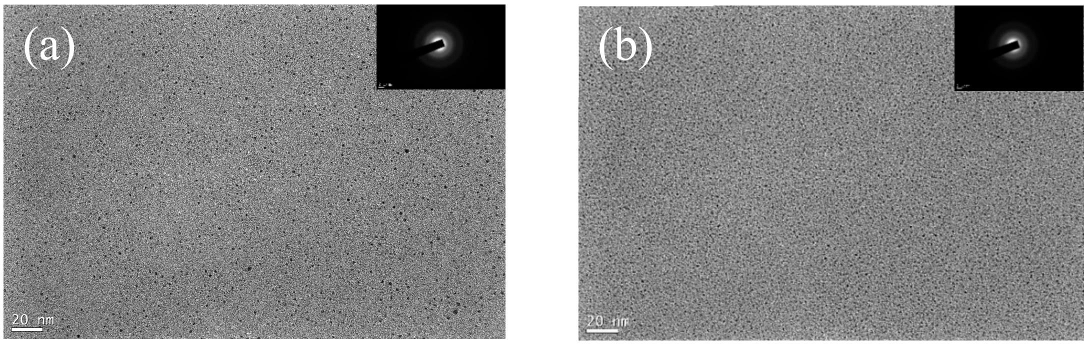

Figure S4. (a) TEM image of $Cs_3Bi_2Cl_9$ QDs, inset shows electron diffraction pattern. (b) TEM image of $Cs_3Bi_2Cl_9$: Ce 30% QDs, inset shows the electron diffraction pattern.

S3. Optical Characterization

UV-vis spectra were taken on a UV-2700, Shimadzu, Japan UV-Vis spectrophotometer, and fluorescence measurements were obtained on a fluorescence spectrophotometer (FL-4600, Hitachi, Japan). The quantum luminous efficiency PLQY of the sample is obtained by the indirect method, and the specific calculation is shown in the following formula:

$$QY_{sample}=\left(\frac{F_{sample}}{F_{ref}}\right)\left(\frac{A_{ref}}{A_{sample}}\right)\left(\frac{n_{sample}^2}{n_{ref}^2}\right)QY_{ref}$$

Among them, "QYsample" is the fluorescence quantum yield of the sample to be tested, QYref is the fluorescence quantum yield of the standard sample, F is the integrated fluorescence intensity, A is the absorbance of the solution, and n is the average refractive index of the solution. The standard reference material is quinine sulfate fluorescence standard solution (25 °C, solvent: 0.1 mol/L H2SO4, PLQY = 57.7%). In order to reduce the reabsorption effect, it is necessary to ensure that the absorbance of the quinine sulfate solution and the solution to be tested are the same at the excitation wavelength. Less than 0.1, at room temperature, the refractive indices of 0.1 mol/L H2SO4 solution and ethanol are approximately 1.3 and 1.1.

S4. XPS Quantitative Analysis

Table S1. XPS binding energies and atomic concentrations of $Cs_3Bi_2Cl_9$ QDs.

	**Cs 3d**	**Bi 4f**	**Cl 2p**
binding energy (eV)	724.5	159.3	198.1
Atomic (%)	1.89	15.03	22.55

Table S2. XPS binding energies and atomic concentrations of $Cs_3Bi_2Cl_9$: Ce 30% QDs.

	Cs 3d	Bi 4f	Cl 2p	Ce 3d
binding energy (eV)	724.5	159.6	198.3	884.9
Atomic (%)	4.97	6.91	20.17	3.01

S5. Time-resolved photoluminescence spectra of $Cs_3Bi_2Cl_9$ and $Cs_3Bi_2Cl_9$: Ce 30% QDs

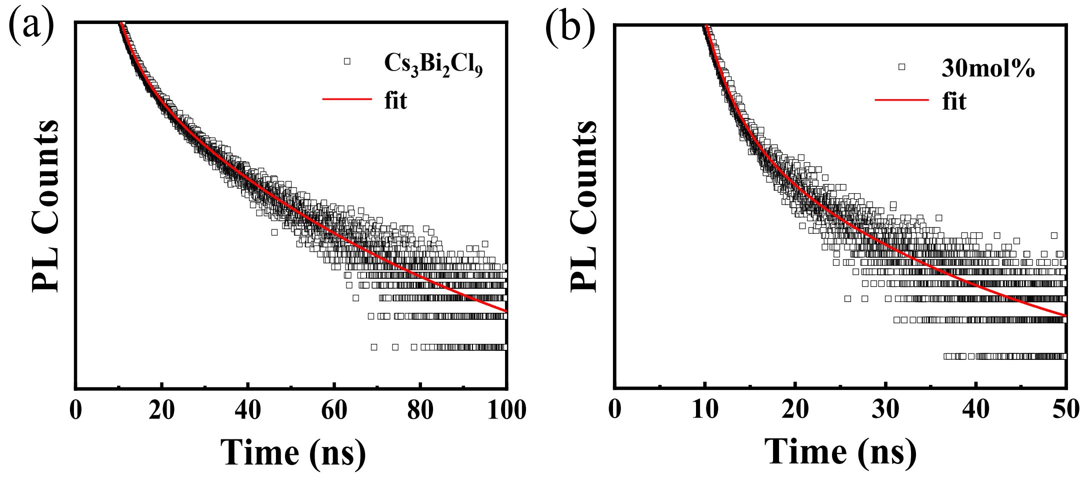

Figure S5. (a). Time-resolved photoluminescence spectra of $Cs_3Bi_2Cl_9$ QDs. (b). Time-resolved photoluminescence spectra of $Cs_3Bi_2Cl_9$: Ce 30% QDs.

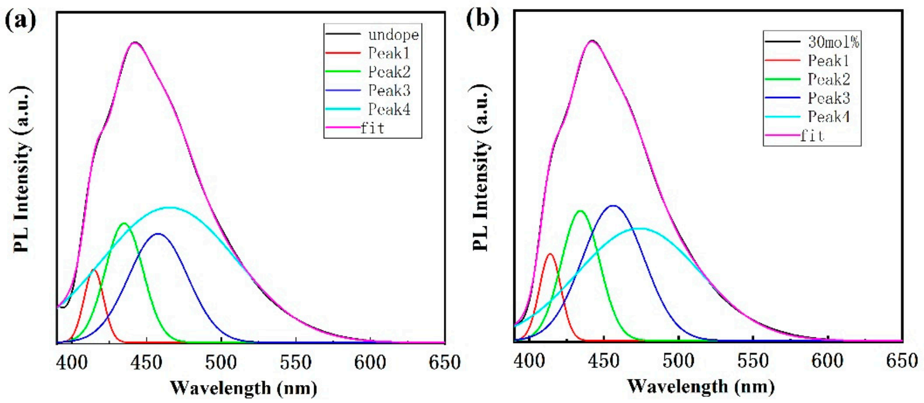

Figure S6 (a) Room-temperature photoluminescence spectrum and related fitting peaks of $Cs_3Bi_2Cl_9$ QDs; (b) room-temperature photoluminescence spectrum and related fitting peaks of $Cs_3Bi_2Cl_9$: Ce 30% QDs.

Table S3. Room-Temperature Fluorescence parameters of fitted peaks for $Cs_3Bi_2Cl_9$ QDs, results normalized.

	**Peak1**	**Peak2**	**Peak3**	**Peak4**
Position (nm)	414	434	457	465
Height	24.1	39.4	36.0	44.7
FHWM (nm)	15.4	29.8	46.7	107.1

Table S4. Room-Temperature Fluorescence parameters of fitted peaks for $Cs_3Bi_2Cl_9$: Ce 30% QDs, results normalized.

	**Peak1**	**Peak2**	**Peak3**	**Peak4**
Position (nm)	414	434	457	473
Height	28.8	43.3	44.8	37.2
FHWM (nm)	17.0	30.3	48.8	97.0

S6.Upconversion fluorescence equipment

The upconversion absorption characteristics of the samples were determined using an optical path consisting of a Ti:sapphire femtosecond pulsed laser (Mira 900S, Coherent), an inverted microscope (Axio Observer A1, Zeiss), a spectrometer (SR-500i-B1, Andor) and other optical components.

S7. Upconversion Fluorescence Properties of $Cs_3Bi_2Cl_9$ and $Cs_3Bi_2Cl_9$: Ce 30% QDs

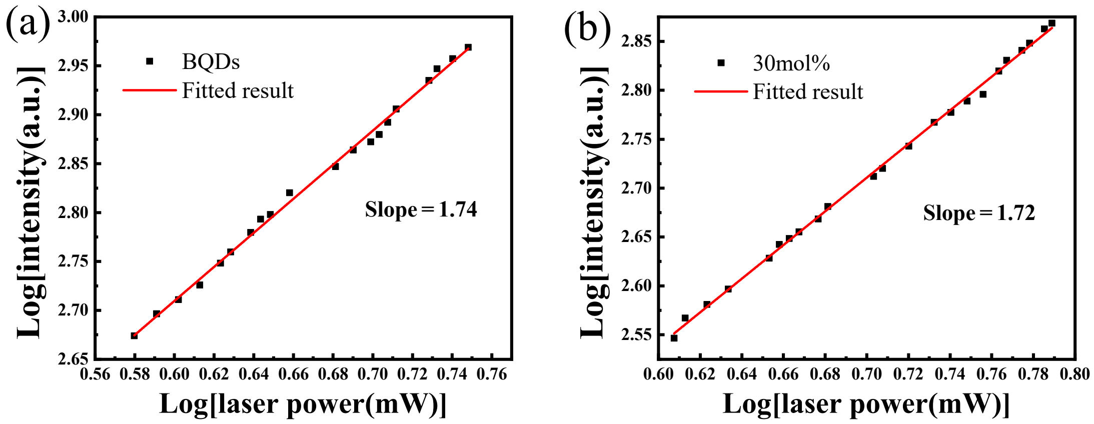

Figure S7. Dependence of upconversion fluorescence intensity of (a) $Cs_3Bi_2Cl_9$ and (b) $Cs_3Bi_2Cl_9$: Ce 30% QDs on excitation power of 750 nm femtosecond pulsed laser

Table S5. Fluorescence quantum efficiencies and upconversion absorption cross sections of $Cs_3Bi_2Cl_9$ and $Cs_3Bi_2Cl_9$: Ce 30% QDs.

**Sample**	**λ_{ex} (nm)**	**λ_{em} (nm)**	**Φ (%)**	**σ_2 (GM)**
R6G	750	553	95	18
$Cs_3Bi_2Cl_9$	750	440	13.28	25.84
$Cs_3Bi_2Cl_9$: Ce 30%	750	440	22.12	24.78

S8. Cell viability and uptake

The relative cell viability of the samples was determined by the MTT method. HepG2 cells were seeded in 96-well plates, and after 12 hours, the original medium was replaced with 100 μL of medium containing the samples, and the concentrations were set to 10 μg/mL, 25 μg/mL, 40 μg/mL, 55 μg/mL, 70 μg/mL, 85 μg/mL mL, 100 μg/mL, 130 μg/mL, 160 μg/mL, 200 μg/mL, after 24 h of incubation, add 15 mL of MTT solution directly to each well. After 4 hours, the waste liquid in the wells was aspirated, and 100 μl of DMSO was added to each well. Finally, cell viability was determined by UV-vis absorbance of the DMSO solution in the wells at a wavelength of 490 nm.

Cellular uptake was determined by using an inductively coupled plasma mass spectrometer (ICP-MS) (ICAP-qc, Thermo Fisher, Germany). HepG2 cells were inoculated in culture flasks, and then the sample solutions prepared with fresh cell

culture medium were respectively replaced (the concentrations of BQDs and (30mol%) BQDs were both 30 μg/mL). Precipitate, count with a cell counting plate, add 2 mL of concentrated nitric acid and 0.5 mL of concentrated hydrochloric acid to the cell suspension, put it in a boiling water bath and keep stirring until the solution is clear and transparent, and then set the volume to 10 mL, thus completing the sample preparation.

S9. Cell imaging

HepG2 cells were seeded on confocal dishes and incubated for 12 hours at 37°C in a humidified atmosphere with 5% CO2. After removing the medium, the cells were incubated with 3 mL of culture medium containing the samples (all samples were at a concentration of 50 μg/mL) for 24 h. Before imaging, cells were washed 3 times with PBS buffer to remove excess perovskite. Upconversion fluorescence images of HepG2 cells were obtained using a multiphoton laser scanning microscope (Nikon A1, Japan) at an excitation wavelength of 750 nm, and the images were processed by Olympus FV31S-SW and Image-J software.

S10. Zeta potential of $Cs_3Bi_2Cl_9$ QDs and $Cs_3Bi_2Cl_9$:Ce (30mol%) QDs

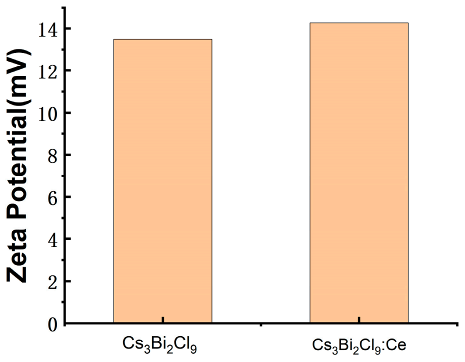

Figure S8. Zeta potential of Cs3Bi2Cl9 QDs and $Cs_3Bi_2Cl_9$:Ce QDs
